# Using the connectome to predict epileptic seizure propagation in the human brain

**DOI:** 10.1186/1471-2202-16-S1-P110

**Published:** 2015-12-18

**Authors:** Timothée Proix, Viktor K Jirsa

**Affiliations:** 1 Aix Marseille Université, Inserm, INS UMR_S 1106, 13005, Marseille, France

## 

Partial seizures in epileptic patients are generated in localized networks, so-called Epileptogenic Zone (EZ), before recruiting other regions, so-called Propagation Zone (PZ) [1]. For drug-resistant patients, surgical resection is sometimes possible. Correctly delineating the extent of the EZ and PZ is critical for a successful surgical resection, in order to remove enough of the epileptogenic tissue to prevent seizures while minimizing the cognitive collateral damages. EZ and PZ extents are evaluated using imaging tools such as M/EEG, MRI, PET and stereotaxic EEG (sEEG). In this modelisation work, we used the large-scale connectome to build a network of neural masses in order to reproduce seizure propagation through the human brain. In particular, we aimed at predicting the propagation of epileptic seizures, i.e. the PZ, using the localization of the EZ.

We preprocessed data obtained from 18 different patients with different types of partial epilepsy. Using MRI and diffusion MRI data, we generated patient-specific connectomes along with cortical surfaces, using different parcellation resolutions. Epileptic dynamics of a single region was based on the Epileptor, a neural mass model able to autonomously generate epileptic seizures [2]. The different regions interacted via a permittivity coupling allowing to reproduce seizures propagation such as observed in sEEG [3]. Using a reduced Epileptor model, we performed a stability analysis at the edge of the seizure onset. We confirmed our results with simulations of the network of Epileptors using The Virtual Brain, a neuroinformatics platform to simulate large-scale dynamics [4]. The analytical prediction of seizure spatial extent correctly reproduces seizure simulations.

**Figure 1:**
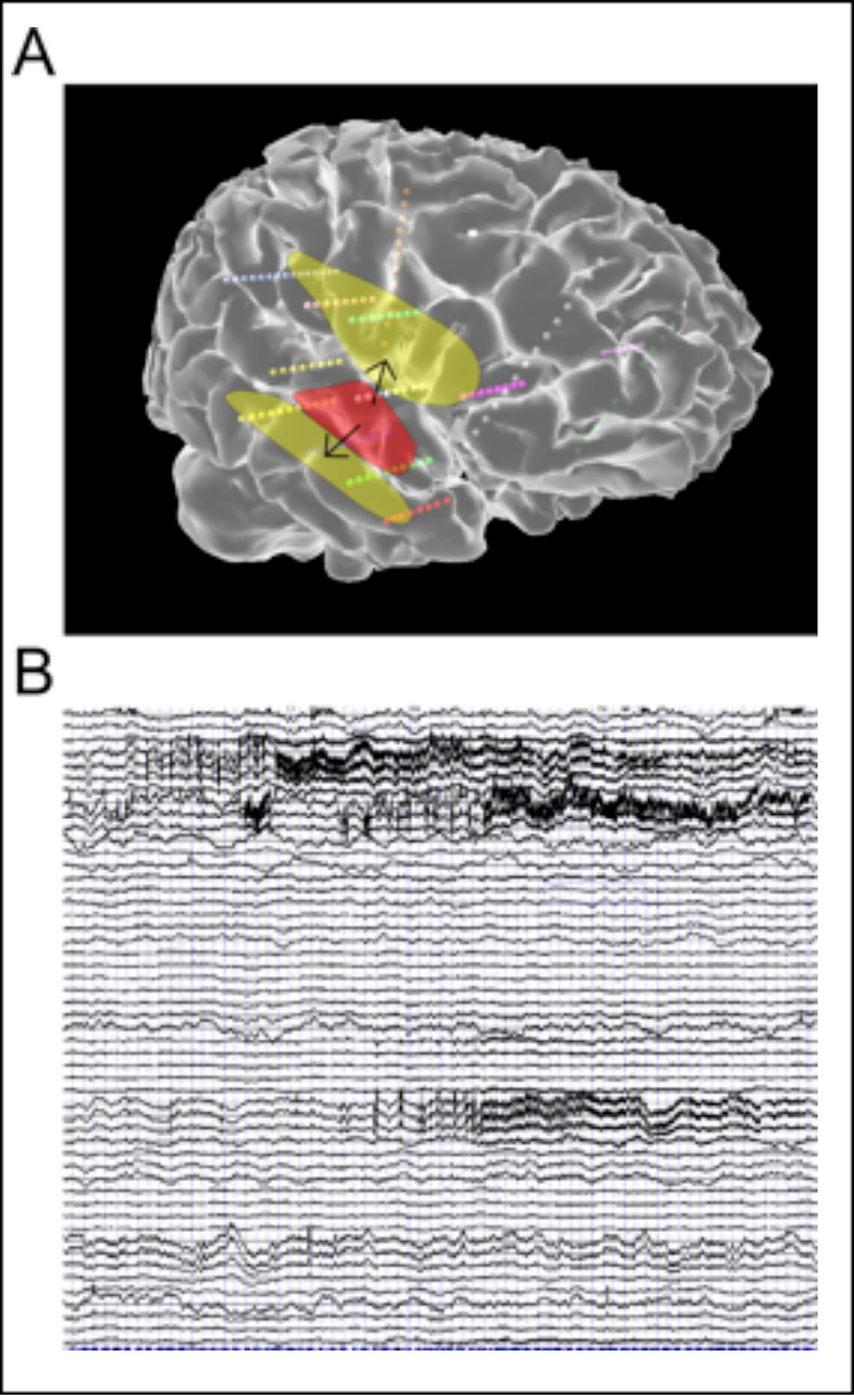
**A. Example of the EZ (red) and the calculated PZ (yellow) displayed on the patient cortical surface, along with sEEG electrodes (small spheres)**. **B**. Corresponding simulated time series.

We systematically predicted the spatial spread of the seizure, i.e. the PZ, for each patient, according to the spatial extent and localization of the EZ such as observed with sEEG, using different global connectivity and excitability parameters. An example of an EZ and PZ along with a simulation of the forward calculation on sEEG electrodes are shown in Figure 1. Our results show a good agreement with clinician predictions, surgery results, and sEEG signals. To confirm the determinant role of the connectome in spatial seizure propagation, we performed several surrogate analysis with other neural mass models (e.g. FitzHugh-Nagumo model), connectivity of control subjects and generic connectivities such as shuffled connectivities, random and small-world networks, again evaluated against clinical data. Real connectomes always performed better than generic connectivities. The connectome particular structure of a patient was often but not always better to predict seizure propagation than connectome of controls.

In conclusion, our results show that large-scale white matter tracts play an important role in the propagation of epileptic seizures. Better understanding of their exact role can help to significantly improve the success rate of surgical resections for epileptic patients.
